# Accelerated Intermittent Theta-Burst Stimulation as a Treatment for Cocaine Use Disorder: A Proof-of-Concept Study

**DOI:** 10.3389/fnins.2019.01147

**Published:** 2019-10-30

**Authors:** Vaughn R. Steele, Andrea M. Maxwell, Thomas J. Ross, Elliot A. Stein, Betty Jo Salmeron

**Affiliations:** ^1^Neuroimaging Research Branch, National Institute on Drug Abuse, Intramural Research Program, National Institutes of Health, Baltimore, MD, United States; ^2^Center on Compulsive Behaviors, Intramural Research Program, National Institutes of Health, Bethesda, MD, United States; ^3^Medical Scientist Training Program, University of Minnesota Medical School, Minneapolis, MN, United States

**Keywords:** cocaine use disorder, intermittent theta-burst stimulation, open-label, accelerated iTBS, dorsolateral prefrontal cortex (DLFPC)

## Abstract

There are no effective treatments for cocaine use disorder (CUD), a chronic, relapsing brain disease characterized by dysregulated circuits related to cue reactivity, reward processing, response inhibition, and executive control. Transcranial magnetic stimulation (TMS) has the potential to modulate circuits and networks implicated in neuropsychiatric disorders, including addiction. Although acute applications of TMS have reduced craving in urine-negative cocaine users, the tolerability and safety of administering accelerated TMS to cocaine-positive individuals is unknown. As such, we performed a proof-of-concept study employing an intermittent theta-burst stimulation (iTBS) protocol in an actively cocaine-using sample. Although our main goal was to assess the tolerability and safety of administering three iTBS sessions daily, we also hypothesized that iTBS would reduce cocaine use in this non-treatment seeking cohort. We recruited 19 individuals with CUD to receive three open-label iTBS sessions per day, with approximately a 60-min interval between sessions, for 10 days over a 2-week period (30 total iTBS sessions). iTBS was delivered to left dorsolateral prefrontal cortex (dlPFC) with neuronavigation guidance. Compliance and safety were assessed throughout the trial. Cocaine use behavior was assessed before, during, and after the intervention and at 1- and 4-week follow-up visits. Of the 335 iTBS sessions applied, 73% were performed on participants with cocaine-positive urine tests. Nine of the 14 participants who initiated treatment received at least 26 of 30 iTBS sessions and returned for the 4-week follow-up visit. These individuals reduced their weekly cocaine consumption by 78% in amount of dollars spent and 70% in days of use relative to pre-iTBS cocaine use patterns. Similarly, individuals reduced their weekly consumption of nicotine, alcohol, and THC, suggesting iTBS modulated a common circuit across drugs of abuse. iTBS was well-tolerated, despite the expected occasional headaches. A single participant developed a transient neurological event of uncertain etiology on iTBS day 9 and cocaine-induced psychosis 2 weeks after discontinuation. It thus appears that accelerated iTBS to left dlPFC administered in active, chronic cocaine users is both feasible and tolerable in actively using cocaine participants with preliminary indications of efficacy in reducing both the amount and frequency of cocaine (and other off target drug) use. The neural underpinnings of these behavioral changes could help in the future development of effective treatment of CUD.

## Introduction

Addiction is a complex neurobiological disease manifested as compulsive substance use in the face of known negative consequences (Volkow et al., 2016). Approximately 25 million Americans use illicit drugs, costing $193 billion annually, in areas such as health care and lost productivity (Ndic, 2010). Nearly 25% of Americans reporting a lifetime drug dependence also report cocaine dependence (Grant et al., 2016). This chronic, relapsing brain disease is characterized by dysregulated circuits related to cue reactivity, reward processing, executive control, and intrinsic network connectivity (Garavan et al., 1999, 2000; Gu et al., 2010; Steele et al., 2014, 2017, 2018a, 2019; Hu et al., 2015; Fedota et al., 2016; Fink et al., 2016; McHugh et al., 2016). Low retention (∼42%) and high relapse (∼70%) rates plague current treatments for cocaine use disorder (CUD; Dutra et al., 2008). As there are no FDA-approved pharmacotherapies for cocaine dependence, it is imperative to identify promising new treatment interventions.

Non-invasive brain stimulation (NIBS), a tool thought to modulate brain circuits, may be a potential treatment approach, as it appears to be efficacious in several neuropsychiatric disorders (Wassermann and Zimmermann, 2012) including addictions (Diana et al., 2017). However, there are only two publications using transcranial magnetic stimulation (TMS) in an open-label fashion for CUD (Terraneo et al., 2016; Sanna et al., 2019). NIBS is designed to transiently stimulate localized cortex (Barker et al., 1985; George et al., 2003; Hallett, 2007; Parkin et al., 2015) and their downstream cortical and subcortical targets. Regions implicated in CUD include dorsolateral prefrontal cortex (dlPFC), anterior cingulate cortex (ACC), inferior frontal gyrus (IFG), orbitofrontal cortex (OFC), striatum, hippocampus, and insula (Jovanovski et al., 2005; Koob and Volkow, 2010; Goldstein and Volkow, 2011; Volkow et al., 2012; Spronk et al., 2013; Steele et al., 2017, 2019). NIBS applied acutely to various circuits has reduced drug craving in nicotine (Li et al., 2013), alcohol (Mishra et al., 2010), heroin (Shen et al., 2016), methamphetamine (Liang et al., 2018), and cocaine (Camprodon et al., 2007; Politi et al., 2008; Hanlon et al., 2015; Terraneo et al., 2016) users.

A potentially viable NIBS application for CUD is intermittent theta-burst stimulation (iTBS; Huang et al., 2005; Bakker et al., 2015). The post-iTBS shift in electrical baseline exceeds the duration measured for repetitive transcranial magnetic stimulation (rTMS; Chistyakov et al., 2010; Holzer and Padberg, 2010; Di Lazzaro et al., 2011) while requiring far fewer pulses and less time to implement, thus allowing for a briefer treatment session, which could improve patient retention. Moreover, a recent non-inferiority assessment showed iTBS to be as effective for treatment-resistant depression as rTMS (Blumberger et al., 2018). Preliminary data from open-label (Camprodon et al., 2007; Politi et al., 2008; Terraneo et al., 2016; Sanna et al., 2019) and single-blind (Hanlon et al., 2015) studies have shown that NIBS can reduce cocaine craving and reduce cocaine usage. However, iTBS in actively using CUD patients, a necessary condition in a treatment environment, needs further exploration. As such, we performed a proof-of-concept study to establish tolerability and feasibility of such an intervention to treat active CUD.

### The Current Study

We recruited non-treatment seeking CUD individuals actively using cocaine at the time they entered the study to receive open-label iTBS targeting left dlPFC. As depression interventions with NIBS elicit positive effects after at least 26–28 sessions (Carpenter et al., 2012; Dunlop et al., 2017), we chose to implement 30 iTBS sessions over a 2-week period. We hypothesized that this intervention would be feasible in cocaine positive participants (i.e., a good safety profile in this population), participants would tolerate iTBS, and participants would reduce their cocaine use (both amount and frequency of use) post-iTBS. A thorough battery of clinical assessments was collected to measure potential off-target effects related to the iTBS intervention, including mood and use of other drugs of abuse.

## Materials and Methods

### Participants

Right-handed individuals (*N* = 19) with moderate to severe CUD, who were non-treatment seeking, provided written, informed consent [6 females, mean (± SEM) age = 47.4 ± 2.0 years, IQ = 95.1 (± 2.7), years of education = 12.5 ± 0.4, years of cocaine use = 23.1 ± 2.6; Table 1]. All procedures were approved by the National Institute on Drug Abuse Institutional Review Board and the Food and Drug Administration (FDA). Exclusion criteria included lifetime history of schizophrenia or bipolar disorder, current moderate to severe SUD on any substance except cocaine, nicotine, or THC, meeting withdrawal or tolerance criteria to alcohol or a sedative/hypnotic/anxiolytic, contraindications to TMS administration such as a history of seizures, medications that lower seizure threshold, first degree relative with a heritable neurological disorder, pregnancy/lactation, tinnitus, hearing loss, history of myocardial infarction, angina, congestive heart failure, cardiomyopathy, stroke or transient ischemic attack, mitral valve prolapse, or any hearing condition currently under medical care, participation in any NIBS session less than 2 weeks prior to consent and NIBS exposure as a treatment within 6 months, or history of head trauma resulting in loss of consciousness lasting over 30 min or sequelae lasting longer than 1 month.

**TABLE 1:** Demographics.

	**All participants *N* = 19 Mean (SEM)**	**Completers *N* = 9 Mean (SEM)**	**Non-completers *N* = 10 Mean (SEM)**
Sex (F/M)	6/13	5/4	1/9
Race (AA/C/+/NR)	14/3/1/1	7/2	7/1/1/1
Ethnicity (H/Not)	1/18	0/9	1/9
Age	47.4 (2.0)	50.8 (1.9)	44.3 (1.9)
IQ	95.1 (2.7)	97.9 (3.9)	93.7 (3.8)
Years of education	12.5 (0.4)	12.9 (0.4)	12.2 (0.8)
Years of cocaine use	23.1 (2.6)	29.4 (2.8)	17.4 (3.4)

*F, female; M, male; AA, African American; C, Caucasian; +, multiracial; NR, not reported; H, Hispanic; Not, not Hispanic; SEM, standard error of the mean. Completers include the nine participants who completed at least 26/30 iTBS sessions and returned for at least one follow-up. Non-completers include the remaining 10 participants who were admitted to the study but either did not complete at least 26/30 iTBS sessions (*N* = 9) or completed 30/30 iTBS sessions but did not return for follow-up (*N* = 1).*

### Study Timeline

**FIGURE 1:**
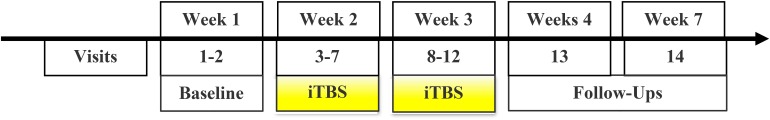
Study timeline: Consent, baseline characterization, and iTBS orientation were implemented over 1–2 visits prior to the initiation of iTBS. Thirty sessions of iTBS were administered over 10 visit days during a 2-week period. Two follow-up appointments were scheduled at 1- and 4-weeks after iTBS.

Following consent, participants completed questionnaires and were assessed for tolerability of the iTBS intervention. Then, 10 days of iTBS were administered over a 2-week period with two sets of five consecutive days scheduled with a 2-day break between weeks. Participants who completed at least 21/30 iTBS sessions were eligible for two follow-up appointments (1- and 4-weeks post-treatment; Figure 1). The first 10 participants were enrolled as inpatients and the last 9 were enrolled as outpatients. Inpatients arrived the night before their first and sixth iTBS day and remained inpatient other than the 2-day break. Upon arrival, participants underwent a search of their person and belongings to ensure abstinence during their inpatient stay. All of the participants who initiated iTBS reported the sessions became more tolerable with number of sessions accumulated. Overall, 335 iTBS sessions were administered with 73% performed on participants with cocaine-positive urine tests.

### Study Attrition

**FIGURE 2:**
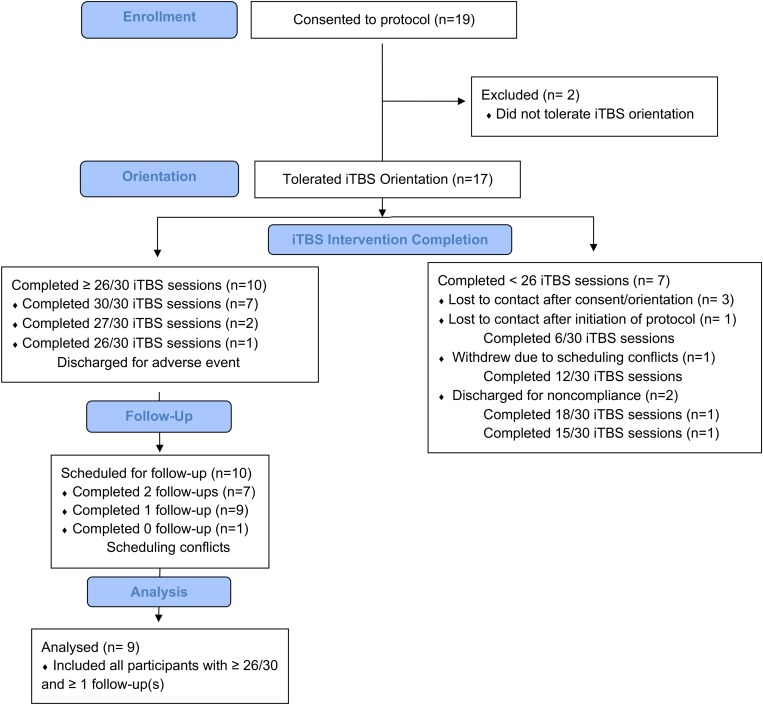
Study attrition: Of the 19 participants consented to the study, 17 of these participants tolerated iTBS orientation, 14 initiated treatment, 10 completed at least 26/30 iTBS sessions. Nine of these 10 returned for at least one follow-up appointment.

Of the 19 participants recruited, 14 initiated iTBS (Figure 2). Of the remaining five participants, two did not tolerate the iTBS and three were lost to contact following consent and iTBS orientation session. Ten of the 14 participants who initiated iTBS received at least 26 of 30 iTBS sessions, two of these did not return for a 1-week follow-up while nine (six as inpatient) returned for a 4-week follow-up session and are defined as “Completers.” No participant returned for the first, but not the second, follow-up session. “Non-Completers” include the remaining 10 participants who were admitted to the study but either did not complete at least 21/30 iTBS sessions (*N* = 9) or completed 30/30 iTBS sessions but did not return for follow-up (*N* = 1). Reasons for not completing include not tolerating iTBS (*N* = 2), lost to contact after consent and prior to initiating iTBS (*N* = 3), lost to contact after completing 2 days of iTBS (*N* = 1), missed a scheduled appointment due to lack of transportation after completing four iTBS days (*N* = 1), discharged after arriving for iTBS day 7 intoxicated (*N* = 1; i.e., non-compliance), and withdrawal due to unwillingness to comply with visitation limits on the inpatient unit (*N* = 1; i.e., non-compliance).

### Clinical Assessments

Self-report and interview-based measures probing mood, motivation, and drug use behavior were collected throughout the protocol and are summarized in Tables 2, 3.

**TABLE 2:** Characterization measurements across treatment timeline.

	**Screening**	**Orientation**	**iTBS day 1**	**iTBS day 10**	**One-week follow-up**	**Four-week follow-up**
Adult ADHD Self Report Scale	*N* = 17	—	—	—	—	—
Attitudes Towards Risk Questionnaire	—	*N* = 18	—	—	*N* = 7	*N* = 9
Addiction Severity Index	*N* = 18	—	—	—	—	—
Beck Anxiety Inventory	—	*N* = 19	—	—	*N* = 9	
Brief Externalizing Inventory	—	*N* = 18	—	—	*N* = 7	*N* = 9
Brief Cocaine Cessation Motivation Assessment	*N* = 19	—	—	—	—	—
Chapman Scales for Physical and Social Anhedonia	—	*N* = 18	—	—	*N* = 7	*N* = 9
Cocaine Craving Questionnaire	—	*N* = 18	*N* = 14	*N* = 9	*N* = 7	*N* = 9
Cocaine Craving Scale	—	*N* = 18	*N* = 14	*N* = 9	*N* = 7	*N* = 9
Cocaine Use, Pattern, and Withdrawal Questionnaire		*N* = 14	—	—	*N* = 7	*N* = 9
Columbia Suicide Severity Scale	—	—	*N* = 14	*N* = 9	—	—
Montgomery-Asberg Depression Rating Scale	—	*N* = 17	*N* = 14	*N* = 9	*N* = 9	—
Nursing Assessment: Hours of Continuous Sleep	—	*N* = 18	*N* = 14	*N* = 9	—	—
Multidimensional Social Contact Circle	—	*N* = 17	—	—	*N* = 7	*N* = 9
Positive and Negative Affect Scale	—	*N* = 19	*N* = 14	*N* = 9	*N* = 7	*N* = 9
Profile of Mood States	—	*N* = 19	*N* = 14	*N* = 9	*N* = 9	
Resting Motor Threshold	—	*N* = 19	*N* = 14	*N* = 9	*N* = 2	*N* = 3
Scale for the Assessment of Positive Symptoms for Cocaine-Induced Psychosis	—	*N* = 7	—	—	*N* = 2	*N* = 3
Snaith – Hamilton Pleasure Scale	—	*N* = 17	—	—	*N* = 7	*N* = 9
Sensation Seeking Scale - V	—	*N* = 17	—	—	*N* = 7	*N* = 9
Temperament and Character Inventory	—	*N* = 17	—	—	—	*N* = 9
Trail Making Task	—	—	*N* = 14	*N* = 9	—	—
Young Mania Rating Scale	—	—	*N* = 14	*N* = 9	—	—

*Characterization measures were collected at different timepoints throughout the screening, iTBS, and follow-up appointments. Of note, due to adjustments in the protocol during data collection, several measures were collected from a limited number of participants. Namely, collection of the “Scale for the Assessment of Positive Symptoms for Cocaine-induced Psychosis” (SAPS-CIP) began following the previously-described unexpected adverse event during which one participant presented with symptoms of psychosis 2 weeks following the termination of treatment. Additionally, collection of the resting motor threshold (RMT) was initiated during the outpatient phase following protocol changes, so only three participants were eligible to receive TMS at their follow-up appointments. The SAPS-CIP was collected from only two participants at the 1-week follow-up because one participant reported no cocaine use since the last day of iTBS, precluding the necessity to administer the questionnaire according to the protocol (see SAPS-CIP in methods). RMT was collected from only two participants at the 1-week follow-up because one participant had received too little sleep the night prior and was ineligible for TMS that day. These versions of assessments were used: Adult ADHD Self-Report Rating Scale (Kessler et al., 2005); Attitudes Towards Risk Questionnaire (Franken et al., 1992); Addiction Severity Index (McLellan et al., 1992); Beck Anxiety Inventory (Beck et al., 1988); Brief Externalizing Inventory (Hall et al., 2007); Brief Cocaine Cessation Motivation Assessment (Boudreaux et al., 2012); Chapman Scales for Physical and Social Anhedonia (Chapman et al., 1976); Cocaine Craving Questionnaire (Tiffany et al., 1993); Cocaine Craving Scale (Weiss et al., 1997); Cocaine Use, Pattern, and Withdrawal Questionnaire (internally generated); Columbia Suicide Severity Scale (Posner et al., 2011); Montgomery-Asberg Depression Rating Scale (Fantino and Moore, 2009); The Multidimensional Social Contact Scale adapted from Linden et al. (2007); Positive and Negative Affect Scale (Watson et al., 1988) was modified by adding an item “Right now I feel detached” because of previous reports of detachment as a potential side effect of TMS (Levkovitz et al., 2007); Profile of Mood States (McNair et al., 1989); Scale for the Assessment of Positive Symptoms for Cocaine-Induced Psychosis (Cubells et al., 2005); Snaith-Hamilton Pleasure Scale (Snaith et al., 1995); Temperament and Character Inventory (Cloninger(ed.), 1994); Time-Line Follow Back; Trail Making Task (Lezak et al., 2004); Young Mania Rating Scale (Young et al., 1978). Dashes indicate “NA,” meaning the questionnaire was not administered at the selected timepoint.*

**TABLE 3:** Self-report and interview-guided measurements across participants.

	**Completers: baseline Mean (SEM)**	**Non-completers: baseline Mean (SEM)**	**Completers: iTBS day 10 Mean (SEM)**	**Completers: 1-week follow-up Mean (SEM)**	**Completers: 4-week follow-up Mean (SEM)**
Attitudes Towards Risk Questionnaire	110.2 (10.2)	111.2 (7.4)	—	112.3 (12.8)	113.2 (7.5)
Adult ADHD Self Report Scale	20.9 (5.1)	17.2 (2.8)	—	—	—
Addiction Severity Index: Drug Composite	0.18 (0.1)	0.1 (0.02)	—	—	—
Beck Anxiety Inventory	3.0 (1.2)	5.5 (2.5)	—	4.0 (1.7)	—
Brief Cocaine Cessation Motivation Assessment: Drive to Quit	20.1 (2.4)	16.8 (2.0)	—	—	—
Brief Externalizing Inventory	375.8 (27.0)	373.3 (26.3)	—	345.9 (30.1)	368.7 (24.4)
Chapman Scales for Physical and Social Anhedonia	32.0 (3.0)	31.7 (3.1)	—	28.7 (4.1)	32.8 (4.2)
Cocaine Craving Questionnaire	178.7 (12.5)	150.1 (11.6)	110.3 (11.2)	107.3 (15.6)	112.8 (12.6)
Cocaine Craving Scale	28.4 (4.7)	26.4 (5.0)	6.6 (3.4)	9.0 (5.6)	20.9 (5.6)
CUP: Mental Withdrawal	6.4 (3.4)	4.2 (1.4)	—	4.7 (0.6)	4.7 (0.8)
CUP: Physical Withdrawal	6.1 (0.5)	6.4 (1.2)	—	6.3 (1.3)	5.7 (0.8)
CUP: Desire to Quit	10.9 (0.7)	11.6 (0.9)	—	11.0 (0.8)	11.57 (1.7)
CUP: Urgency to Use	7.9 (1.3)	1.9 (0.8)	—	3.9 (1.1)	3.6 (1.0)
CUP: Negative Drive to Use	13.9 (1.1)	12.6 (2.8)	—	12.1 (1.3)	11.9 (1.4)
CUP: Positive Drive to Use	8.8 (1.4)	10.0 (2.2)	—	7.0 (1.5)	8.1 (0.7)
CUP: Social Factors to Use	7.8 (1.0)	6.2 (0.9)	—	7.4 (1.04)	8.7 (0.7)
CUP: Avoidance of people/places associations with use	3.9 (0.6)	4.4 (0.6)	—	5.7 (0.56)	5.9 (0.53)
Columbia Suicide Severity Scale	0.7 (0.6)	0.00 (0)	0.00 (0)	—	—
Montgomery-Asberg Depression Rating Scale	3.9 (41.6)	1.0 (0.8)	1.0 (1.0)	0.7 (0.4)	—
Multidimensional Social Contact Circle	20.2 (5.7)	19.2 (3.8)	—	22.0 (5.6)	15.4 (4.5)
Nursing Assessment: Hours of Continuous Sleep	7.9 (0.3)	7.2 (0.4)	6.7 (0.5)	6.4 (0.9)	6.8 (0.5)
PANAS: Detachment	1.11 (0.1)	1.4 (0.2)	1.25 (0.25)	1.4 (0.4)	1.63 (0.3)
POMS: Fatigue	2.6 (1.2)	2.2 (0.8)	4.0 (1.4)	2.3 (1.0)	—
POMS: Confusion	1.7 (0.7)	1.9 (1.2)	2.4 (1.1)	2.0 (0.7)	—
POMS: Anger-Hostility	1.1 (0.8)	2.3 (1.0)	3.5 (2.0)	2.1 (1.3)	—
POMS: Tension	5.6 (1.0)	7.2 (1.7)	5.4 (1.3)	3.9 (1.1)	—
POMS: Depression	5.7 (2.2)	6.0 (6.3)	7.4 (2.8)	5.2 (1.5)	—
POMS: Vigor	16.2 (7.3)	14.8 (2.2)	16.5 (1.03)	16.6 (2.2)	—
Resting Motor Threshold	62.9 (3.1)	54.1 (4.3)	62.1 (1.8)	55.0 (8.0)	61.0 (6.0)
Scale for the Assessment of Positive Symptoms for Cocaine-Induced Psychosis	5.7 (3.8)	2.25 (0.2)	—	7.5 (5.5)	0.67 (0.67)
Sensation Seeking Scale - V	19.7 (2.7)	17.9 (1.1)	—	21.7 (3.4)	19.1 (2.7)
Snaith – Hamilton Pleasure Scale	0.44 (0.2)	1.1 (0.4)	—	0.9 (0.3)	0.44 (0.2)
TCI: Novelty	23.1 (21.6)	21.8 (1.0)	—	—	21.1 (2.0)
TCI: Harm Avoidance	13.1 (1.5)	13.8 (2.9)	—	—	14.2 (2.)
TCI: Reward	13.3 (1.5)	13.8 (1.1)	—	—	13.4 (1.7)
TCI: Persistence	6.3 (0.4)	4.8 (0.7)	—	—	6.9 (0.5)
TCI: Self-Directedness	26.7 (2.1)	29.8 (2.2)	—	—	26.0 (2.1)
TCI: Cooperativeness	31.8 (1.7)	31.0 (2.3)	—	—	32.7 (1.3)
TCI: Self Transcendence	17.8 (1.9)	11.9 (2.4)	—	—	19.7 (2.1)
TMT: Trial A (Errors/Duration in seconds)	0.44 (0.2) 30.8 (3.3)	0.0 (0.0) 24.8 (1.7)	0.38 (0.3) 25 (2.0)	—	—
TMT: Trial B (Errors/Duration in seconds)	0.44 (0.2) 54.0 (3.9)	0.80 (0.4) 51.2 (1.7)	0.75 (0.3) 53.5 (3.6)	—	—
Young Mania Rating Scale	0.11 (0.1)	0.00 (10.6)	0.38 (0.2)	—	—

*CUP, Cocaine Use, Pattern, and Withdrawal Questionnaire; POMS, profile of mood states; SD, standard deviation; TCI, Temperament and Character Inventory; TMT, Trail Making Task. Dashes indicate “NA,” meaning the questionnaire was not administered at the select timepoint. Completers include the nine participants who completed at least 26/30 iTBS sessions and returned for at least one follow-up. Non-completers include the remaining 10 participants who were admitted to the study but either did not complete at least 26/30 iTBS sessions (*N* = 9) or completed 30/30 iTBS sessions but did not return for follow-up (*N* = 1).*

Several additional measures generated internally were also implemented. The Cocaine-Induced Psychosis: Screener (CIP: Screener) was designed by one of us (BJS) for efficient assessment of cocaine-induced psychosis and was used to assess changes, relative to baseline, in symptoms of psychosis throughout the protocol. This assessment was administered on all study days after the baseline visit if the participant reported cocaine use since the last visit. Any change from baseline triggered administration of the full Scale of Positive Symptoms for Cocaine-Induced Psychosis (SAP-CIP; Cubells et al., 2005). The iTBS Monitoring Questionnaire is a 13-item interview-based yes/no questionnaire assessing side effects of TMS (e.g., headaches, nausea, seizure). The Positive and Negative Affect Scale (PANAS; Watson et al., 1988) was modified by adding an item “Right now I feel detached” because previous reports of detachment have been reported as a potential side effect of TMS (Levkovitz et al., 2007). The Cocaine Use, Pattern, and Withdrawal Questionnaire was designed (BJS) to assess the general pattern of use and withdrawal of cocaine using participants.

At the beginning of each study day, participants received a nursing assessment, comprised of vital signs (e.g., blood pressure, heart rate, pulse oximetry, respiration, temperature), hours of sleep, observed urine sample for toxicology, urine pregnancy tests, and TMS safety screen. Time and date of last food intake, drug and alcohol use, and prescription medication use were also collected at each nursing assessment. Participants were not required to be cocaine-negative prior to iTBS treatment but did need to pass a neuromotor assessment (Heishman et al., 1996) indicating no signs of acute intoxication. Two hours post-TMS, vitals (blood pressure and heart rate) were assessed.

### Monitoring Cognitive and Affective Changes

At the suggestion of the FDA, several measures were collected to specifically assess cognitive and affective changes potentially linked to chronic iTBS administration in an actively cocaine using sample. The timing of these measures was designed to assess potential detrimental off-target effects of iTBS. Several assessments were collected daily, before and after iTBS administration: iTBS monitoring questionnaire, the modified PANAS (Watson et al., 1988), Cocaine Craving Questionnaire (CCQ; Tiffany et al., 1993), and the Cocaine Craving Scale (CCS; Weiss et al., 1997). Assessments of mood disturbance and cognition were collected at the beginning and end of the 2-week iTBS administration: Columbia Suicide Severity Scale – (C-SSS; Posner et al., 2011), Montgomery-Asberg Depression Rating Scale (MADRS; Fantino and Moore, 2009), Profile of Mood States (POMS; McNair et al., 1989), Trail Making Task (TMT; Lezak et al., 2004), and Young Mania Rating Scale (YMRS; Young et al., 1978). The Time-line Follow Back (TLFB) was collected at the beginning of each study day whenever the participant was not an inpatient to assess ongoing drug use (in addition to daily urine toxicology).

### Transcranial Magnetic Stimulation

#### Equipment

A MagVenture MagPro X100 with MagOption Stimulator was used throughout the study. Two MagVenture figure-of-8 coils were used: CB 60 was used for single pulses and the and A/P Coil was used for iTBS administration. Participant-specific motor hotspot and left dlPFC treatment locations were saved via the neuronavigation system Brainsight (Rouge Research, Quebec, Canada). Left dlPFC was located using the software Beam_F3 Locator, which allows localization of the F3 electrode location from the 10–20 EEG system for prefrontal TMS applications (Beam et al., 2009). Adaptive PEST, a non-parametric algorithm for estimating TMS motor threshold (Borckardt et al., 2006), was used to determine resting motor threshold (RMT; described below). All TMS sessions occurred with the participant seated in a comfortable chair, with the ability to recline if needed. A chinrest and head support 60 cm from a computer screen were used during iTBS administration for participant comfort and to ensure similar stimuli viewing experience among participants.

#### Orientation

During the orientation day, we identified motor hotspot, determined RMT, collected a recruitment curve, and assessed the tolerability of iTBS. Motor hotspot was defined as the region of the left motor cortex that reliably elicited movement of the contralateral abductor pollicis brevis (APB) muscle and/or an associated motor-evoked potential (MEP). TMS stimulation that elicited any movement in the contralateral hand and/or a MEP of at least 50 microvolts was counted as a positive response. The recruitment curve (i.e., dose/response curve) consisted of 42 total pulses applied to the motor hotspot while MEPs were recorded. Six pulses were administered at each of seven intensities ranging from 90 to 120% of RMT over about 5 min with jittered interstimulus interval (5–10 s). The MagVenture A/P coil was positioned for iTBS on the scalp using the Brainsight neuronavigation location identified previously with Beam_F3. Ramping of the stimulator output starting about 20 percentage points below RMT allowed a gradual increase of intensity as tolerated by the participant. When participants affirmed ramping between trains until they received two trains at their RMT, the toleration was deemed successful. If the iTBS administration was too painful (i.e., intolerable), participants could cease administration at any point. Generally, the stimulator was ramped by five points between each iTBS train until reaching RMT.

#### Intermittent Theta-Burst Stimulation

We implemented an accelerated iTBS treatment protocol, which entailed three iTBS sessions per day, with at least a 60-min interval between sessions, for 10 days yielding 30 overall iTBS sessions. Each iTBS session consisted of 600 pulses in 50 Hz bursts of three pulses, separated by 200 ms (i.e., a 5 Hz frequency) for 2 s, followed by 8 s of no pulses over about 190 s (Huang et al., 2005). The stimulator was ‘ramped’ (described above) to 100% of RMT for each session. Prior to each iTBS study day, the CB60 coil was used to confirm motor hotspot and determine the RMT. We collected recruitment curves before and after every iTBS session of the 1st, 5th, 6th, and 10th treatment day. TMS recruitment curves were acquired for two participants at the first follow-up and three participants at the second follow-up. Recruitment curves were not acquired at all follow-up visits because collection of this measure was added to the protocol at the onset of the outpatient phase. One participant was not able to receive TMS [i.e., resting motor threshold (RMT) determination and recruitment curve] at the first follow-up because he reported too little sleep (<5 h) the night prior. During each iTBS session, participants viewed cocaine-related pictures and were instructed to actively inhibit their cocaine craving using individualized strategies previously discussed with the study physician based on a cognitive-behavioral therapy intervention for CUD. Pictures (gathered internally and from collaborators) were each presented for 30 s with a 1-s fixation cross between images. TMS-safe goggles were provided for individuals requiring prescription lenses.

### Data Analysis

Linear Mixed Models were performed in *R* to test our hypotheses that participants would reduce both the amount and frequency of cocaine use after iTBS, relative to baseline. Only Completers were included in the analyses. Statistical significance was judged against a threshold of *p* < 0.05. Because this was a proof-of-concept study with a small sample size, statistical tests were applied only to primary outcomes of amount and frequency of drug use. Qualitative assessment of trends is discussed for other measures. Although MEPs were recorded during recruitment curves, technical issues (clipped and noisy signals) during data collection preclude analysis of these data.

## Results

### Drug Use Behavior

**FIGURE 3:**
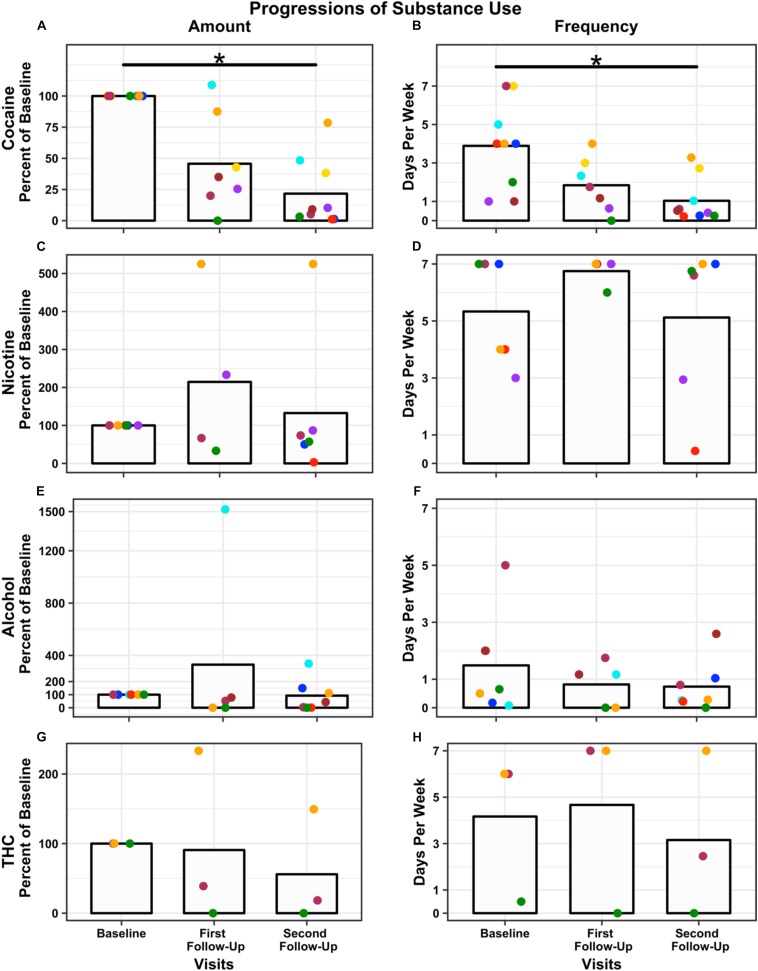
Change in drug use over time: Amount (percent used relative to baseline) and frequency (days used per week) are presented for each time point, baseline, first follow-up at 1-week, and second follow-up at 4-week post-iTBS. Amount of use is plotted for each substance: cocaine **(A)**; nicotine **(C)**; alcohol **(E)**, and THC **(G)**. Frequency of use is plotted for each substance: cocaine **(B)**; nicotine **(D)**; alcohol **(F)**, and THC **(H)**. *N* = 9 used cocaine, *N* = 5 used nicotine, *N* = 7 used alcohol, and *N* = 3 used THC. Bars represent the average across participants at each time point (note two participants did not return for the first follow-up). Each dot represents a participant; dot color remains consistent within participant across time points and substance. Significance was assessed for cocaine only and not other substance use. Both amount and frequency of cocaine use significantly decreased post-iTBS ^∗^*p* < 0.001. Results are presented graphically for qualitative assessment only.

There were no qualitative differences in cocaine use at baseline between Completers and Non-Completers based on the TLFB. At the 4-week post-iTBS follow-up, the nine Completers reduced the amount of money (in US Dollars) spent on weekly cocaine consumption from $197 (*SD* = $115) at baseline to $30 (*SD* = $40) at the second follow-up, a 78% reduction (first follow-up reduction = 54%, *M* = $61, *SD* = $45), *F*(2,14) = 17.54, *p* < 0.001 (Figure 3A) and reduced the number of days of use from 4 (*SD* = 2) days per week at baseline to 1 (*SD* = 1) days per week at the second follow-up, a 70% reduction (first follow-up reduction = 44%, *M* = 2 days, *SD* = 1 days), *F*(2,14) = 12.91, *p* < 0.001, relative to pre-iTBS (Figure 3B). Similarly, other drug use generally decreased (Figures 3C–H). Specifically, participants reduced their cigarettes per week (baseline: *M* = 79, *SD* = 105; first follow-up: *M* = 25, *SD* = 22; second follow-up: *M* = 40, *SD* = 53. Note a heavy smoker did not return for the first follow-up) by 4%, number of alcohol drinks consumed (baseline: *M* = 10, *SD* = 9; first follow-up: *M* = 12, *SD* = 15; second follow-up: *M* < 1, *SD* < 1) per week by 8%, and both amount (baseline: *M* = 9, *SD* = 12; first follow-up: *M* = 4, *SD* = 5; second follow-up: *M* = 7, *SD* = 10) and frequency of marijuana joints (baseline: *M* = 4, *SD* = 3; first follow-up: *M* = 5, *SD* = 4; second follow-up: *M* = 3, *SD* = 4) per week by 44 and 90%, respectively. Two individuals increased their nicotine, alcohol, and/or THC use. One participant increased nicotine and THC use relative to baseline because of personal struggles that occurred during the study (i.e., separation from his wife and child). The second participant substantially increased his alcohol consumption relative to baseline because of reported binge drinking while on a date after iTBS. Neither increase in use appeared to be compensation for a reduction in cocaine use nor directly related to participating in this study.

Interestingly, in addition to the reported changes in use, participants also reported a change in their relationship with cocaine post-iTBS. Specifically, they spontaneously reported a reduced drive to use cocaine, an ability to stop using after initiating use (i.e., reduction in compulsive drug use) and, notably, reported they were unable to get as ‘high’ relative to pre-iTBS. One participant reported using threefold her normal amount of cocaine in an attempt to replicate her previous cocaine ‘high’ but was unsuccessful and was then able to stop using. Another participant returned for an unrelated study 1-year post-iTBS and reported her reduced cocaine use had persisted. She reported only using a limited amount on Friday and Saturday nights, would skip using for family events, her drive to use was reduced, and she was able to maintain the full-time employment she secured post-iTBS, whereas prior to iTBS she used cocaine daily and was not regularly employed.

### Other Effects

Self-report craving measured with the CCS and CCQ decreased during the 2-weeks of iTBS administration and then increased at the follow-up visits, although they did not return to pre-iTBS levels. There were also qualitative changes in urgency to use (slight decrease), increase avoidance of people/places associated with use, decrease MADRS, and decrease SAP-CIP from baseline to follow-up visits. Other measures remained unchanged in the Completers following iTBS (Table 3). All participants who completed at least 4 days of iTBS exhibited improved mood. Many spontaneously reported a shift toward a positive outlook. Daily RMT remained consistent throughout the study (Table 4).

**TABLE 4:** Resting motor threshold throughout the study.

**Participant**	**Baseline**	**iTBS day 1**	**iTBS day 2**	**iTBS day 3**	**iTBS day 4**	**iTBS day 5**	**iTBS day 6**	**iTBS day 7**	**iTBS day 8**	**iTBS day 9**	**iTBS day 10**	**One-week follow-up**	**Four-week follow-up**
04	58	55	63	56	57	60	57	59	62	51	60	—	—
05	51	53	59	49	54	58	51	51	53	59	62	—	—
07	74	76	79	78	79	77	73	74	77	76	69	—	—
08	59	60	67	52	52	60	51	56	56	52	59	—	—
09	54	53	46	47	40	42	47	46	49	43	—	—	—
10	77	73	76	77	76	74	80	74	70	79	57	—	—
16	57	52	59	57	57	57	58	59	62	—	57	47	54
17	72	71	68	—	78	65	68	71	71	68	63	—	73
18	64	62	62	61	63	68	57	68	61	62	70	63	56

*Resting motor threshold (RMT) was recorded at each study visit throughout the study. Recording RMT at follow-up visits was implemented only in the outpatient phase of the study. RMT was collected from only two of three eligible participants at the first follow-up because one participant reported too little sleep (<5 h) the night prior and was ineligible for TMS that day. Numbers represent percent of maximum stimulator output of the MagVenture MagPro X100 with MagOption used in this study. Dashes indicate “NA,” meaning the RMT was not collected at the given timepoint.*

### Adverse Events and iTBS Monitoring Questionnaire Results

Across all participants over the entire protocol, there were no unexpected, serious adverse events. Nine of the 14 participants who began iTBS sessions experienced at least one headache, usually beginning during or shortly after iTBS but a few in the evening after sessions were completed. Four experienced three or more headaches throughout the protocol. Most were mild and resolved without intervention. Seven headaches were reported after iTBS that required a single dose of acetaminophen; two participants each had two headaches and one participant had three. One participant experienced sudden pain around her eyes about an hour after completing her final iTBS session on day 7, which was accompanied by muscle twitching around the left eye and a dark spot in her left lateral peripheral vision which resolved in a few minutes. One experienced muscle soreness in the right forearm at the start of the second week of iTBS which resolved in 1 day. No negative side-effects in cognitive and affective assessments were reported or observed after iTBS. No participant experienced any signs of mania or suicidality.

One participant experienced two adverse events of note. After completing 26/30 iTBS sessions during the inpatient phase, the participant reported right-hand supination/pronation at the wrist 10–15 min following the iTBS session. These rhythmic hand movements continued for about 3 min, reduced to one twitch every 3–5 min, and dissipated within 1 h. Her participation in the iTBS portion of the protocol was terminated. This was classified as a neurological event of unknown etiology. Two weeks following the iTBS termination, this same participant reported visual illusions and tactile hallucinations after using cocaine. These symptoms likely reflected cocaine-induced psychosis, a common occurrence in chronic cocaine users (Vergara-Moragues et al., 2014) but one this participant had never previously experienced prior to study participation. Her symptoms developed slowly over several days but cleared promptly with a single dose of olanzapine. This participant prompted the inclusion of the SAPS-CIP and CIP: Screener in the outpatient phase of the study. Further details can be found in a previously published case report (Steele et al., 2018b).

The iTBS Monitoring Questionnaire revealed no seizures, fainting, difficulties speaking or understanding speech, or impairment of thought. One participant noted brief, mild dizziness after the second iTBS session on the fifth day. One participant reported wakening suddenly with a jerk once a night after iTBS days 3, 4, and 5, something she had not experienced previously. One participant noted some intermittent tinnitus after completing all sessions.

## Discussion

This accelerated iTBS protocol was well-tolerated with a good safety profile in an actively-using, non-treatment seeking CUD population. The most frequently reported side effect was the occasional mild headache, which remitted either spontaneously or following acetaminophen administration. Individuals who completed the protocol reduced their weekly cocaine consumption by 78% in amount of dollars spent and 70% in days of use relative to pre-iTBS cocaine use patterns. Similarly, Completers reported modest reductions in their weekly consumption of nicotine, alcohol, and THC. Much of this polydrug usage was not associated with cocaine use, suggesting that iTBS may have modulated a common neural circuit engaged across drugs of abuse. The safety profile was good, although a single participant developed a transient neurological event of uncertain etiology on iTBS day 9 and cocaine-induced psychosis 2-weeks after iTBS termination (Steele et al., 2018b).

The anecdotal improvements in mood were striking in their similarity across individuals along with reduced compulsive cocaine use post-iTBS. Participants also reported a reduction in short-term craving during the protocol, similar to previous reports of NIBS in cocaine using populations (Camprodon et al., 2007; Politi et al., 2008; Hanlon et al., 2015; Terraneo et al., 2016; Sanna et al., 2019). However, these were short-lived in that craving increased at the 4-week follow-up visit, though without returning to the higher baseline levels.

Although no attempt was made in this open-label study to measure neural circuit alterations, the behavioral changes reported herein are likely attributable to left dlPFC iTBS affecting dysregulated circuits related to CUD. Broad fMRI activity changes (Fox et al., 2012) and increases in DA release in the caudate nucleus (Strafella et al., 2001; Keck et al., 2002) have been reported with left dlPFC stimulation. In fact, network connectivity between the dlPFC and the anterior cingulate cortex [ACC; a dysregulated hub in both depression and addiction and part of a functional network predictive of CUD treatment outcomes (Hong et al., 2009; Steele et al., 2018a)] is normalized with this intervention in major depression (Fox et al., 2012), supporting network malleability with NIBS. The cognitive and affective dysregulations seen in SUD are associated with neural alterations in the ACC, insula, and/or striatum and may be susceptible to left dlPFC NIBS modulation (Fox et al., 2012). Together, these data suggest that stimulation of the left dlPFC is a potential intervention in addiction (Diana et al., 2017).

Transcranial magnetic stimulation treatment targets should be related to clinically significant outcomes (e.g., relapse, treatment completion) and neural circuitry known to be dysregulated in addiction. During our iTBS administration, participants were instructed to actively inhibit their cocaine craving while viewing cocaine-related pictures. Perhaps, the iTBS and behavioral interventions influenced executive control leading to reduction in cocaine use post-iTBS. Executive control, dysregulated in SUD, requires circuits including dlPFC, ACC, IFG, OFC, striatum, hippocampus, and insula (Jovanovski et al., 2005; Koob and Volkow, 2010; Goldstein and Volkow, 2011; Volkow et al., 2012; Spronk et al., 2013; Steele et al., 2017, 2019). Both, event-related potential (ERP) measures of executive control, specifically error-processing (Marhe et al., 2013; Marhe and Franken, 2014; Steele et al., 2014; Fink et al., 2016) thought to originate in the ACC (van Veen and Carter, 2002; Edwards et al., 2012), and fMRI measures (Luo et al., 2013; Steele et al., 2018a) predict drug treatment outcomes. Bolstered post-error processing in ERP measures (Steele et al., 2014) and stronger functional connectivity between ACC and striatum, amygdala, and hippocampus (Steele et al., 2018a) is predictive of treatment completion. Enhancing executive control (i.e., increasing post-error processing) and functional connectivity of dysregulated circuits via iTBS while inhibition of craving could provide a viable treatment target for CUD.

### Limitations

Although our findings are promising for use of iTBS as a treatment for CUD, there are study limitations to consider. First, this was an open-label study. All participants knew they would receive active iTBS, posing the risk for a placebo effect, as with any intervention. Additionally, participants actively participated in craving suppression during each of the three daily iTBS sessions, so our results may also relate to the intensive practice of craving reduction, independent of iTBS. As a proof-of-concept study, our goal was to assess feasibility and tolerability of iTBS as a potential intervention in actively using cocaine dependent individuals, not to differentiate the effects of active and sham stimulation during craving suppression. Second, we report a small number or participants (*N* = 9) who completed a substantial number of iTBS sessions and returned for a follow-up visit. Based on this limited number of observations, strong conclusions cannot be drawn. Nonetheless, Completers did reduce their substance use post-iTBS with largely similar anecdotal accounts of changes in their interactions with cocaine, warranting further study with a larger sample to better understand this phenomenon. Recall that subjects were explicitly recruited as non-treatment seekers in a non-treatment intervention-although they were told that their cocaine use might change after TMS. Third, we had a limited duration of follow up as our primary concern was to establish the feasibility of undertaking a large, sham controlled study; our only 1-year follow up was serendipitous. Finally, the neural underpinnings of behavioral changes reported here remain untested; uncovering these should benefit future iTBS applications as a SUD treatment. Based on these pilot data, we have now begun a large-scale, double-blind, sham-controlled trial of iTBS as an experimental treatment for CUD with longitudinal fMRI and follow-up (NCT02927236). Because, substance users are known to have dysregulated cue reactivity, reward processing, executive control, and intrinsic network connectivity (Garavan et al., 1999, 2000; Gu et al., 2010; Hu et al., 2015; Fedota et al., 2016; Steele et al., 2017, 2019), we will assess these cognitive processes and measure their related neural mechanisms before and after acute and chronic application of iTBS. The study is specifically designed to measure the trajectory of neuroplastic change induced by an iTBS intervention and how that relates to drug use behavior.

## Conclusion

In this open-label, proof-of-concept study of accelerated iTBS in CUD, we measured and reported the safety and tolerability of this intervention as well as multiple clinical assessments relevant to SUD treatment. Even in this cohort of non-treatment seeking cocaine dependent individuals, substance use decreased both for hypothesized targeted cocaine and also for ‘off-target’ use of other substances, including nicotine, alcohol, and THC along with improved mood. Adverse side-effects were limited, and we did not observe seizures, fainting, difficulties speaking or understanding speech, or impairment of thought, all of which are occasionally reported following NIBS interventions. We offer three main take-away messages. First, individuals with active CUD can tolerate accelerated iTBS and adhere to an intense 2-week, 30 session schedule. Second, iTBS applied at 100% of RMT to actively using cocaine users did not result in a concerning rate of negative side effects in this small sample. Third, as an open-label, small sized study, no strong conclusions can be made. Generally, however, we believe this report lays the groundwork for larger studies in active cocaine using CUD individuals to assess neuroplastic changes interrogated with neuroimaging techniques to better understand those circuits affected by iTBS, in what manner, the longevity of such effects, and their relationship to drug use behavior (cf. Ekhtiari et al., 2019).

## Data Availability Statement

The datasets generated for this study are available on request to the corresponding author.

## Ethics Statement

The studies involving human participants were reviewed and approved by the National Institute on Drug Abuse Institutional Review Board and the Food and Drug Administration. The patients/participants provided their written informed consent to participate in this study.

## Author Contributions

VS, ES, and BS conceived the study approach. VS, AM, and BS collected the data. VS devised and implemented the analytical approach, and performed the data analysis. VS and AM drafted the manuscript. All authors contributed to the interpretation of the data, provided critical revisions, and approved the final version of the manuscript submitted for publication.

## Conflict of Interest

The authors declare that the research was conducted in the absence of any commercial or financial relationships that could be construed as a potential conflict of interest.
